# Representative Chemical Formulas of Cadmium Selenide Quantum Dots Determined with a Combination of Mass Spectrometry and Nuclear Magnetic Resonance

**DOI:** 10.3390/nano16140895

**Published:** 2026-07-22

**Authors:** Nickie Tiwari, Igor Fedin

**Affiliations:** Department of Chemistry and Biochemistry, The University of Alabama, Tuscaloosa, AL 35487, USA

**Keywords:** mass spectrometry, quantum dots, molecular formula, surface ligands

## Abstract

Matrix-assisted laser desorption/ionization time-of-flight (MALDI-TOF) mass spectrometry (MS) has the potential to characterize the size and composition of quantum dots (QDs). However, examples of its usage in the literature are limited. In this study, we perform an in-depth characterization of the size and composition of the CdSe QD system using a combination of MALDI MS and nuclear magnetic resonance (NMR). Using these techniques, we determine the size and composition of a series of wurtzite and zincblende CdSe QDs. The composition ranges from 176 to 1202 CdSe units per QD. With this information, we construct a sizing curve for the QDs and compare it with sizing curves from the literature obtained using other techniques such as transmission electron microscopy (TEM) and small-angle X-ray scattering (SAXS). These results will be insightful for the community developing complex multilayer QD compositions for photonic applications.

## 1. Introduction

Colloidal quantum dots (QDs) have emerged as promising candidates for biomedical imaging, photovoltaics, sensors, light displays, and catalysis [1,2,3,4,5]. QDs are semiconductor nanocrystals that have size-dependent band gaps due to quantum confinement [6,7]. They have distinct advantages such as tunable emission, sharp spectral features, and solution processability [8,9]. The size of QDs is used to calculate various parameters, including QD concentration and extinction coefficient, that are needed for core–shell synthesis and device fabrication [10,11]. Moreover, the optoelectronic properties of QDs are highly influenced by their surface ligands [12]. Ligands passivate the QD surface from trap states [13] and can be used to fine-tune properties such as charge transfer and dispersion in aqueous media, which are paramount for biomedical and photovoltaic applications [14]. For this reason, there has been significant interest in studying ligand binding to the QD surface [15,16,17,18]. As a result, determining the size and composition of QDs accurately and efficiently is essential. Matrix-assisted laser desorption/ionization time-of-flight (MALDI-TOF) mass spectrometry (MS) can be an effective tool for characterizing QD composition and size in tandem with other techniques, such as transmission electron microscopy (TEM) [19] and small-angle X-ray scattering (SAXS) [20]. In the literature, MALDI-TOF has been used to determine the molecular ion peak of various QD systems, including CdSe clusters [21,22,23,24] and CdSe [25,26], CdTe [27], CdS [28,29], Cd_3_P_2_ [30], ZnS [31], and InP QDs. Typically, the size is determined from the position of the molecular ion peak. In addition, Xie et al. utilized MALDI with NMR and optical absorption spectroscopy to determine the composition of InP QDs [32]. Although MS has proven useful, its use as a characterization technique for QDs remains fairly limited. For example, CdSe is a well-studied system, but few studies have used MALDI MS. Seo et al. used an electron-withdrawing fluorinated porphyrin (TPFP) as a matrix to lower the ionization thresholds of CdSe QDs and prevent ligand detachment [25]. Using the TPFP matrix, they achieved a signal at lower laser thresholds while preserving the ligands, enabling them to quantify the size and composition of various CdSe QDs [25]. We hope to further characterize the composition of CdSe QDs using MS and NMR. In this study, we synthesize wurtzite (wz) and zincblende (zb-) CdSe QDs of a series of sizes. We use a combination of MALDI-MS, NMR, and optical absorption spectroscopy to determine the size and composition of the QDs. MALDI-MS will be used to determine the molecular weight of the QDs, while NMR will be used to quantify the ligand-to-QD ratio [32]. From the molecular weight and ligand-to-QD ratio, the composition and size of the QDs will be determined. We note that QDs [25] and atomically defined clusters [33] typically exhibit broad peaks in the mass spectrum, primarily due to size and elemental isotopic distributions. As a result, there will be uncertainty when determining the molecular ion peak, which we account for in error propagation. Finally, using the size determined from MS, we construct a sizing curve for wz- and zb-CdSe QDs and compare it to the published sizing curves based on TEM and SAXS.

## 2. Materials and Methods

Cadmium oxide (CdO, 99.999%, Sigma-Aldrich, Burlington, MA, USA), cadmium nitrate tetrahydrate (99.997%, Sigma-Aldrich), oleic acid (OA, 90%, Alfa Aesar, Ward Hill, MA, USA), myristic acid (99%, Sigma-Aldrich), selenium powder (200 mesh, 99.998%, Alfa Aesar), selenium (shot, 2–6 mm, 99.998%, Alfa Aesar), trioctylphosphine (TOP, 97%, Strem, Newburyport, MA, USA), 1-octadecene (ODE, 90%, Alfa Aesar), ethanol (200 proof), and toluene (anhydrous 99.8%, Sigma-Aldrich) were used as received.

Cadmium myristate was produced by adding a solution of cadmium nitrate into a solution of sodium myristate in methanol. The resulting precipitate was filtered, washed with methanol, and dried. To prepare a 0.50 M Cd oleate stock, 2.568 g (20.00 mmol) of CdO is mixed with 20 mL oleic acid (OA) and 20 mL octadecene (ODE) in a three-neck flask, degassed at room temperature, then heated under vacuum to 120 °C and stirred until the CdO fully dissolved and the mixture became clear; the solution was then brought under nitrogen and cooled to form a white solid. To prepare 2.0 M trioctylphosphine selenide (TOP-Se) solution, 3.158 g (40.00 mmol) of Se shot was stirred with 20 mL TOP in the glovebox under mild heating overnight until the selenium completely dissolved.

Wz-CdSe QDs were synthesized based on the procedure by Landry et al. [33] 0.80 mL of 0.50 M cadmium oleate (0.40 mmol) was degassed at 110 °C in 6.0 mL of 1-octadecene (ODE). 0.10 mL of 2.0 M trioctylphosphine selenide (0.20 mmol TOP-Se) was injected at 290 °C under N_2_ from 1 to 5 min to form QDs of the desired size. Larger QDs (with the first exciton peak from 2.09 to 2.04 eV) were synthesized based on the procedure by Kozlov et al. [34] 0.20 mL of 0.50 M cadmium oleate (0.10 mmol) in 6.0 mL ODE was degassed at 110 °C. 0.10 mL of 2.0 M TOP-Se (0.20 mmol) was injected at 290 °C. 1.0 mL of TOP was injected dropwise after 40 s over 20 s. After the core reached the band-edge exciton absorption peak at 548 nm, a solution of 0.375 mL of ODE, 0.50 mL of 0.50 M cadmium oleate (0.25 mmol), and 0.125 mL of 2.0 M TOP-Se (0.25 mmol) was continuously injected at 5.0 mL/h.

Zb-CdSe QDs capped with oleate ligands were synthesized using a modified procedure by Capek et al. [35] 0.40 mmol of cadmium oleate and 0.40 mmol of selenium powder were degassed at 110 °C in 6.0 mL ODE. The mixture was heated to 235 °C under N_2_ for 1 to 5 min to form QDs of the desired size. Zb-CdSe QDs capped with myristate ligands were synthesized using a modified procedure by Cao et al. [36] 0.40 mmol of cadmium myristate and 0.40 mmol of selenium dioxide were degassed at 110 °C in 6.0 mL of ODE. The mixture was heated to 180 °C under N_2_ for 1 to 5 min to form QDs of the desired size. A minimal amount of oleic acid was injected to ensure colloidal stability.

Matrix-assisted laser desorption ionization (MALDI) time-of-flight (TOF) mass spectrometry (MS) experiments were conducted on a Bruker rapifleX mass spectrometer (Billerica, MA, USA) equipped with a Bruker scanning smartbeam 3D laser of 355 nm wavelength (≥100 μJ per pulse) in the high mass region based on the procedure in our previous work with cadmium phosphide clusters [30]. For mass calibration in the reflectron mode, peptides angiotensin I, somatostatin-28, and ubiquitin at 1 mg mL^−1^ in water were mixed with 2-nitrophloroglucinol (2-NPG, 50 mM, 50/50 acetonitrile/water, 0.1% trifluoroacetic acid) matrix at 1:1:1:3 volume ratio, and 1 μL was spotted on a stainless steel MALDI target. A solution of QDs in toluene was mixed with 10 mM 5,10,15,20-tetrakis-(pentafluorophenyl) porphyrin (TPFP) in chloroform in a 1:1 volume ratio, with 1.0 μL applied on the target and dried [25]. The MS spectra were acquired in linear positive ion mode for the high-mass region (2–200 kDa) with Bruker Flex Control 4.0 and analyzed with Flex Analysis 4.0.

The ligand concentration for the QD samples was determined using the internal standard method. We dried the QD sample under vacuum after measuring its absorbance and dispersed it in deuterated chloroform at a known standard concentration. We used THF as the internal standard and integrated the peak at 3.58 ppm (4H). For the wz- and zb-CdSe QDs, we integrated the olefin proton peak at a chemical shift of 5.36 ppm (2H). The details of the calculations of the ligand concentrations are in the Supplementary Materials.

Transmission electron microscopy (TEM) measurements of CdSe QD samples were performed at the University of Alabama using a Talos 200i microscope (Thermo Fisher Scientific Inc., Waltham, MA, USA). Dilute solutions of QDs were deposited on lacey carbon copper grids and dried. Quantitative ^1^H NMR was performed on a Bruker Avance 500 spectrometer (Billerica, MA, USA) using tetrahydrofuran (THF) as the internal standard.

All inductively coupled plasma—mass spectrometry (ICP-MS) measurements were carried out by the Geological Element Analysis Laboratory at the University of Alabama using an Agilent 8900 Triple Quadrupole ICP-MS (Santa Clara, CA, USA).

## 3. Results

### 3.1. Ionization and Composition of Quantum Dots from MALDI-MS

QDs can be ionized through two processes in MALDI-TOF MS [25]. In the first process, the matrix is photoionized, producing a radical cation. If the QD has a lower ionization energy than the matrix, the QDs are ionized to a radical cation. In the second process, QDs can be excited by the laser, producing an exciton. The neutral matrix can then extract the electron from the excited QD state, producing the QD radical cation.

To estimate the size and composition of the QDs, we followed the procedure detailed by L. Xie et al. for InP [32]. From the peak molecular weight, we determined the extinction coefficient of the QDs (ε), which is provided in the Supporting Information (Table S1). The concentration of the ligands in the QD samples was determined with NMR by integrating the characteristic olefin protons of oleic acid against an internal standard, tetrahydrofuran (THF). Oleate ligands are X-type ligands that are covalently bonded to the QD surface [37,38]. Because of that, ligand loss from drying and exchanging solvents is unlikely. It should be noted that bound ligands exhibit broadened peaks in NMR while free ligands exhibit sharp peaks. For this reason, we integrated the broad peak to quantify the bound ligand concentration. From the concentrations of the QDs and the ligands, we determined the ligand per QD ratio (*N*_L_). Using the ligand per QD ratio, we determined the mass of the inorganic part (*M*_i_) and the number of CdSe units per QD (*U*_CdSe_), which can be seen in Equations (1) and (2).

(1) $$M_{i}=M-N_{L}\times M_{oleate}-\frac{N_{L}}{2}\times A_{Cd},$$

(2) $$U_{CdSe}=\frac{M_{i}}{M_{CdSe}},$$

Finally, we determined the QD diameter (*D*) using the bulk density of CdSe (Equation (3)).

(3) $$D=\sqrt[{3}]{\frac{\left(\frac{U_{CdSe}}{\rho_{CdSe}}\times 6\right)}{\pi }},$$

Through this method, the main source of error is the broad molecular ion peak of the QDs. The primary reasons for the broadening are the size distribution of the analyzed batch and possible fragmentation during analysis. Additionally, there is an isotopic distribution in CdSe. Cadmium has six stable isotopes, while selenium has five, both of which contribute to a complex isotope distribution in the MS peaks. For this reason, we used the peak mass for each batch and the half-width at half maximum as the uncertainty (∆M).

The uncertainty in the number of units of CdSe (*U*_CdSe_) and diameter (*D*) is summarized in Equations (4) and (5). The contributors to the uncertainty are the molecular ion peak (*M*), the mass of QD samples (*m*) to determine the extinction coefficient, the ligand per QD (*N*_L_), and the inorganic mass (*M*_i_). The constant *C* = 83,943 results from the molecular masses of Cd and oleate. A more detailed calculation is provided in Supplementary Materials.

(4) $$∆U_{CdSe}=U_{CdSe}\times \sqrt{\frac{\left[C\times \left(N_{L}^{2}\right)\times \left[{\left(\frac{∆m}{m}\right)}^{2}+\left({\left(\frac{∆M}{M}\right)}^{2}\right)\right]\right]+{∆M}^{2}}{M_{i}}},$$

(5) $$∆D=\frac{1}{3}D\times \sqrt{\frac{\left[C\times \left(N_{L}^{2}\right)\times \left[{\left(\frac{∆m}{m}\right)}^{2}+\left({\left(\frac{∆M}{M}\right)}^{2}\right)\right]\right]+{∆M}^{2}}{M_{i}}},$$

### 3.2. MALDI MS of Wurtzite CdSe Quantum Dots

**Figure 1:**
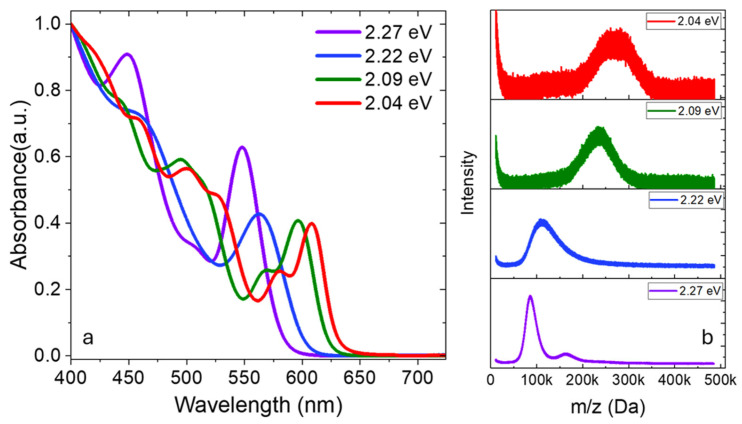
(**a**) Absorption spectra of wz-CdSe quantum dots (QDs) of four different sizes (marked as energies of the first excitonic peaks). (**b**) The corresponding matrix-assisted laser desorption/ionization time-of-flight (MALDI-TOF) mass spectra of the four samples of wz-CdSe QDs.

First, we synthesized four batches of wz-CdSe QDs of different sizes with the first exciton absorption wavelength from 546 to 608 nm (energy in the range from 2.27 to 2.04 eV) (Figure 1a). The crystal structure was confirmed with powder X-ray diffraction (XRD) (Figure S6), and we further analyzed only those samples where we saw the characteristic (102) peak of wurtzite. We performed MALDI-MS to determine the molecular weight of these QDs (Figure 1b). We observed the expected shift from smaller to larger peak masses, along with broadening of the molecular band as QD size increased. The average mass determined from the mass peak ranged from 88 to 269 kDa across the four samples (Table 1).

The highest-absorption-energy QDs (2.27 eV) had an average mass of 88 kDa, corresponding to 392 ± 69 CdSe units with an average size of 3.4 ± 0.2 nm. We should note that for the smallest QDs, there is a small peak at 169 kDa, likely corresponding to trace amounts of larger QDs in the sample that are not detected in the absorption spectra. This further highlights the sensitivity of MALDI MS. Meanwhile, the lowest absorption energy was 2.04 eV, and the corresponding QDs had an average mass of 248 kDa, which projects 1297 ± 303 CdSe units with an average size of 5.1 ± 0.4 nm. The sizes determined from MS-NMR agreed with the TEM sizes when uncertainty was considered. Importantly, the uncertainty of the QD size propagated from the hwhm of the mass spectra was smaller than the uncertainty of TEM measurements.

### 3.3. MALDI MS of Zincblende CdSe Quantum Dots

**Figure 2:**
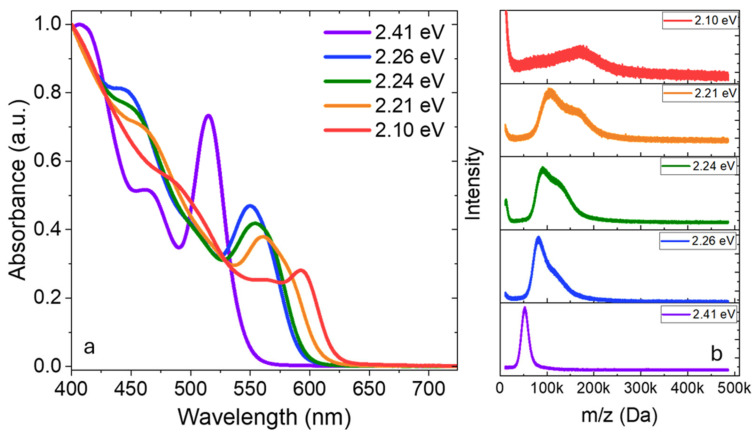
(**a**) Absorption spectra of zb-CdSe QDs of four different sizes (marked as energies of the first excitonic peaks). (**b**) The corresponding MALDI-TOF mass spectra of the four samples of zb-CdSe QDs.

We synthesized five batches of zb-CdSe QDs with oleate ligands. The absorption spectra of zb-CdSe QDs showed the first exciton absorption energies ranging from 2.41 to 2.10 eV (Figure 2a). The crystal structure was confirmed with the XRD (Figure S6). As with wz-CdSe QDs, the average mass of the peaks increased with decreasing absorption energy for all samples of zb-CdSe and ranged from 53 kDa to 156 kDa (Figure 2b, Table 2). MS-TEM revealed an increase in the number of CdSe units and in size, with a decreased energy gap, for all samples. For instance, the highest energy band gap QD of 2.41 eV has an average mass of 53 kDa that corresponds 179 ± 48 CdSe units with an average size of 2.7 ± 0.2 nm, whereas the smallest energy band gap QDs of 2.1 eV have an average mass of 156 kDa that corresponds to 732 ± 248 CdSe units with an average size of 4.3 ± 0.5 nm. We note that, for QDs with first excitonic energies ranging from 2.21–2.26 eV, we observed bimodal peaks in the mass spectrum, which could correspond to the polydisperse nature of the QDs. This is further supported by the broad exciton peaks in the absorption spectra. When the MALDI size projections are compared to TEM, the MS sizes agreed with TEM. We were unable to image the 2.47 eV band gap QDs due to the small size. This shows an instance where MALDI-MS could have an advantage over TEM when characterizing small QDs by their molecular weight. We also synthesized five batches of zb-CdSe QDs capped with myristate ligands. The MS, absorption spectra, and compositions are provided in the Supplementary Materials (Figure S7 and Table S2). The band gaps ranged from 2.47 eV to 1.99 eV. The myristate-capped QDs exhibited similar trends to the oleate-capped QDs. As the band gap decreased, the myristate QDs increased in average mass, CdSe units, and size. For the myristate zb-CdSe QDs with a 2.47 eV band gap, the average mass was 43 kDa, corresponding to 156 ± 79 CdSe units with an average size of 2.6 ± 0.4 nm. Similarly, for the myristate zb-CdSe QDs with a 1.99 eV band gap, the average mass was 316 kDa, corresponding to 1293 ± 364 CdSe units with an average size of 5.2 ± 0.5 nm. Ultimately, the size and composition, as projected by the MS, were in close agreement for the myristate- and oleate-capped QDs.

## 4. Discussion

Typically in mass spectrometry, the composition of the analyzed compound is assigned solely by the molecular ion peak for a variety of materials such as gold nanoparticles [39] and peptides [40]. The composition is determined by precisely matching the mass of the components of the material to the molecular ion peak mass. Due to the size distribution of the QDs and the complex isotope distribution in CdSe QDs, which broadens the peaks, assigning the formula to this level of precision is challenging. However, we tentatively assigned the composition of one of our samples based on peak masses and compared it with the composition determined by MS and NMR. For the wz-CdSe QDs that absorbed at 2.27 eV, we obtained a peak mass of 87,939.8 Da, which was used for calculations. Assuming that the QD is charge balanced, we assigned a composition of (CdSe)_396_[Cd(C_17_H_33_CO_2_)_2_]_18_ that predicts an average mass of 87,938.6 Da, making the difference 1.2 Da. With the use of a combination of MS and NMR, the composition predicted was (CdSe)_392_[Cd(C_17_H_33_CO_2_)_2_]_19_. In this instance, the compositions predicted by both methods seem to align when uncertainty is taken into account.

**Figure 3:**
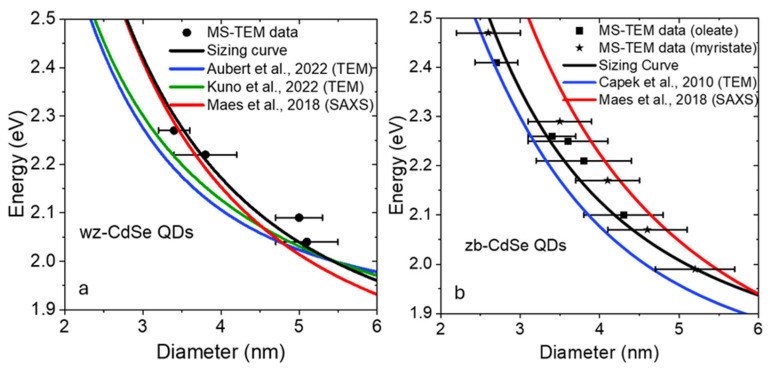
(**a**) MS-NMR size data and sizing curve for wz-CdSe QDs in comparison to published sizing curves using other techniques by Aubert et al. [41], Kuno et al. [43], Maes et al. [20]. (**b**) MS-NMR size data and sizing curve for zb-CdSe QDs in comparison to published sizing curves using other techniques by Capek et al. [35], Maes at al. [20].

We constructed a sizing curve using MS-NMR and compared it to other techniques in the literature, such as TEM and SAXS (Figure 3). We fitted our data using the linearized equation developed by Z. Hens et al. [41]. In the literature, X. Peng et al. [42,43] and P. Mulvaney et al. [44] formed sizing curves for wz-CdSe QDs using TEM, while Z. Hens formed a sizing curve for wz-CdSe QDs using SAXS [20]. For zb-CdSe QDs, Z. Hens et al. formed a sizing curve using TEM [35] and SAXS. For the wz-CdSe QDs, at higher energies, the combination of MALDI MS predicts larger sizes for QDs when compared to TEM. Then, there is an interval where sizing curves converge for both TEM and MS-NMR for lower energies. At this point, MS-NMR predicts slightly smaller QD sizes than TEM at lower energies.

Compared with SAXS, the combination of MS and NMR predicts larger QD sizes across all energies. However, the difference in sizes increases at lower energies compared to higher energies. For zb-CdSe QDs, MS-NMR predicts larger sizes for QDs when compared to TEM for all energies. In contrast to SAXS, MS-NMR predicts smaller QD sizes. However, at lower energies, the MS-NMR and SAXS sizing curves start to converge. It should be noted that Seo et al. also saw that MALDI predicted larger QD sizes than reported TEM sizing curves for their CdSe QD samples [25]. In addition, we assessed the goodness of fit for the MS-NMR sizing technique in relation to SAXS by calculating the χ^2^ and forming residual plots (Table S3). For wz- and zb-CdSe QDs, the χ^2^ for the MS-NMR fit, was 0.06 and 0.10, while the χ^2^ for the SAXS fit was 0.18 and 0.05. The residuals ranged from 0.1 to −0.5 for the MS-NMR fit, while the residuals ranged from 0.05 to −0.55 for the SAXS fit. Overall, the comparable χ^2^ and residuals support the effectiveness of MALDI-MS in relation to other characterization techniques. We acknowledge that the broad peaks in MALDI contribute to errors in the average mass of QDs, which in turn could cause deviations from other sizing techniques. However, for smaller QDs, TEM imaging is difficult, which could also contribute to deviations at higher energies. Moreover, QD interactions with the solvent, as well as polydisperse size distributions, can complicate SAXS analysis and contribute to deviations [45,46]. As a result, MS-NMR is an effective tool to characterize size and composition in tandem with these techniques.

## 5. Conclusions

The combination of MS and NMR proved to be a reliable tool for characterizing the composition and size of QDs using the molecular ion band. Using a combination of MS and NMR, we determined the molecular formulas of wz- and zb-CdSe QDs containing 180 to 1300 CdSe units and constructed sizing curves that agreed with the published ones. Ultimately, this study further supports the effectiveness of MS as a characterization technique that can be used in tandem with TEM and SAXS to elucidate the size and structure of QDs. Going forward, this in-depth MS-NMR characterization can be expanded to other QD systems such as lead chalcogenides (PbS, PbSe). However, because PbS QDs are more prone to multiple excitons, multiple charged ion peaks can be present in the mass spectrum [25]. As a result, to study other QD systems, the intrinsic properties of QD material need to be taken into consideration when optimizing conditions needed for MALDI-MS.

## Figures and Tables

**Table 1:** Summary of the molecular weight from MALDI MS, the composition of wz-CdSe QDs, the deduced QD size, and the QD size measured in TEM.

First-Exciton Absorption Energy	Peak Position in MALDI MS (Da)	Deduced Representative Composition	Calculated Size of the Core	Measured Size in TEM
2.27 eV	87,940 ± 13,036	(CdSe)_392±69_[Cd(C_17_H_33_CO_2_)_2_]_19_	3.4 ± 0.2 nm	3.1 ± 0.5 nm
2.22 eV	117,388 ± 29,689	(CdSe)_515±157_[Cd(C_17_H_33_CO_2_)_2_]_28_	3.8 ± 0.4 nm	3.5 ± 0.7 nm
2.09 eV	233,476 ± 37,748	(CdSe)_1178±197_[Cd(C_17_H_33_CO_2_)_2_]_12_	5.0 ± 0.3 nm	4.2 ± 0.7 nm
2.04 eV	268,528 ± 57,806	(CdSe)_1297±303_[Cd(C_17_H_33_CO_2_)_2_]_30_	5.1 ± 0.4 nm	4.5 ± 0.6 nm

**Table 2:** Summary of the molecular weight from MALDI MS, the composition of zb-CdSe QDs, the deduced QD size, and the QD size measured in TEM.

First-Exciton Absorption Energy	Peak Position in MALDI MS (Da)	Deduced Representative Composition	Calculated Size of the Core	Measured Size in TEM
2.41 eV	53,133 ± 8703	(CdSe)_179±48_[Cd(C_17_H_33_CO_2_)_2_]_28_	2.7 ± 0.2 nm	
2.26 eV	87,614 ± 16,879	(CdSe)_355±90_[Cd(C_17_H_33_CO_2_)_2_]_29_	3.4 ± 0.3 nm	3.1 ± 0.7 nm
2.24 eV	107,882 ± 32,714	(CdSe)_415±175_[Cd(C_17_H_33_CO_2_)_2_]_42_	3.6 ± 0.5 nm	3.3 ± 0.6 nm
2.21 eV	124,128 ± 43,029	(CdSe)_507±229_[Cd(C_17_H_33_CO_2_)_2_]_40_	3.8 ± 0.6 nm	3.5 ± 0.8 nm
2.10 eV	156,340 ± 47,212	(CdSe)_732±248_[Cd(C_17_H_33_CO_2_)_2_]_24_	4.3 ± 0.5 nm	4.1 ± 0.5 nm

## Data Availability

All data are available upon request.

## Supplementary Materials

The following supporting information can be downloaded at https://www.mdpi.com/article/10.3390/nano16140895/s1. Figure S1: Linearized fits to the Hens sizing equation of (a) wz-CdSe (b) zb-CdSe; Figure S2: TEM Images of wz-CdSe QDs absorbing at (a) 2.27 eV, (b) 2.22 eV, (c) 2.09 eV, (d) 2.04 eV; Figure S3: TEM Images of zb-CdSe QDs absorbing at (a) 2.26 eV, (b) 2.24 eV, (c) 2.21 eV, (d) 2.10 eV; Figure S4: NMR of w-CdSe QDs with the internal standard THF; Figure S5: NMR of zb-CdSe QDs with internal standard THF; Figure S6: Powder XRD of (a) zb-CdSe QDs absorbing at 2.21eV and (b) wz-CdSe QDs absorbing at 2.09 eV with the corresponding Miller indices; Figure S7: (a) Absorption spectra of five samples of myristate-capped zb-CdSe QDs of different absorption energies of the first exciton (1.99–2.47 eV). (b) The MALDI-TOF MS of the five samples of zb-CdSe QDs; Figure S8: Residual plots of (a) MS-NMR fit and (b) SAXS fit for wz- and zb-CdSe QDs; Table S1: Extinction coefficients of wurtzite (wz) and zinc blende (zb) CdSe QDs; Table S2: A summary of the molecular weight from MALDI, the composition of myristate-capped zb-CdSe QDs, and the MALDI-MS size; Table S3: Summarizing the χ^2^ for the MALDI and SAXS fits for their respective data sets for wz and zb CdSe QDs; Table S4: Comparison of the Cd:Se ratio for wz-CdSe QDs determined by MALDI-MS and ICP-MS; Table S5: Comparison of the Cd:Se ratio for zb-CdSe QDs determined by MALDI and ICP-MS. References [32,41] are cited in the supplementary materials.

## Abbreviations

The following abbreviations are used in this manuscript:

MS	Mass spectrometry
MALDI	Matrix-assisted laser desorption/ionization
NMR	Nuclear magnetic resonance
TEM	Transmission electron microscopy (microscope, micrograph)
