# Supplementary material for: Representative Chemical Formulas of Cadmium Selenide Quantum Dots Determined with a Combination of Mass Spectrometry and Nuclear Magnetic Resonance
Source: Nanomaterials (Basel). 2026 Jul 22;16(14):895. doi: 10.3390/nano16140895 (PMC13415139; doi:10.3390/nano16140895)
Supplement: Supplementary file 1 [file nanomaterials-16-00895-s001.zip › nanomaterials-4425448-supplementary.pdf]

Supporting Information

Representative Chemical Formulas of Cadmium Selenide Quantum Dots Determined with a Combination of Mass Spectrometry and Nuclear Magnetic Resonance

Nickie Tiwari[1] and Igor Fedin[1]*

1. Department of Chemistry and Biochemistry, The University of Alabama, Tuscaloosa, AL, 35487, United States

E-mail: ifedin@ua.edu

Determining the average mass from MALDI-MS (*M*):

The mass spectrum of each QD sample was taken three times. Then, the masses from the three trials were averaged and called the average mass (*M*), which was used in the calculations.

Uncertainty: The half-width at half maximum for the uncertainty of the average mass (*M*).

Extinction Coefficient (ε):

To determine the size and composition, we followed the procedure presented by Bawendi et al.[1] We measured the MS of wz- and zb-CdSe QDs and determined the molecular mass as described above. To determine the extinction coefficient of the QDs, we weighed the dried samples. Then, we dispersed the QDs in a known volume and measured the absorbance. Using the following equation S1, we determined the extinction coefficient (ε) for the 1st exciton wavelength for the QDs.

$$\varepsilon = \frac{A}{\left(\left(\frac{m}{M}\right)/V\right)} \qquad \text{(S1)}$$

Where A is the absorbance at the 1st exciton wavelength, *m* is the mass of the QD sample, *M* is the average molecular weight of the QD from MS, and *V* is the volume of the solution. The corresponding uncertainty is given by (the relative errors in *A* and *V* are negligible compared to *m* and *M*):

$$\frac{\Delta\varepsilon}{\varepsilon} = \sqrt{\left(\frac{\Delta m}{m}\right)^2 + \left(\frac{\Delta M}{M}\right)^2} \qquad \text{(S2)}$$

QD Concentration:

The QD concentration [QD] was determined by measuring the sample's absorbance and using equation S2.

$$A = \varepsilon b * [\mathrm{QD}] \qquad \text{(S3)}$$

Here *A* is the absorbance at the first exciton wavelength, *b* is the path length of the cuvette (1 mm), and ε is the calculated extinction coefficient.

$$\text{Uncertainty:} \frac{\Delta[\mathrm{QD}]}{[\mathrm{QD}]} = \sqrt{\left(\frac{\Delta\varepsilon}{\varepsilon}\right)^2} = \frac{\Delta\varepsilon}{\varepsilon} \qquad \text{(S4)}$$

Ligand Concentration:

The ligand concentration for the QD samples was determined using the internal standard method. We dried the QD sample under vacuum after measuring its absorbance and dispersed it in deuterated chloroform at a known standard concentration. We used THF as the internal standard and integrated the peak at a chemical shift of 3.58 ppm (4H). For the wz- and zb-CdSe QDs, we integrated the olefin proton peak at a chemical shift of 5.36 ppm (2H). The ligand concentration was calculated using equations 3 and 4.

$$[\mathrm{L}] = \frac{\text{normalized area analayte} \times \text{standard concentration}}{\text{normalized area standard}} \qquad \text{(S5)}$$

$$\text{normalized area} = \frac{\text{peak integral}}{\text{number of protons}} \qquad \text{(S6)}$$

Ligand to QD ratio:

The ligand to QD ratio (N_L) was calculated as:

$$N_{\mathrm{L}} = \frac{[\mathrm{L}]}{[\mathrm{QD}]} \quad \text{(S7)}$$

Uncertainty: $\frac{\Delta N_{\mathrm{L}}}{N_L} = \sqrt{\left(\frac{\Delta[\mathrm{QD}]}{[\mathrm{QD}]}\right)^2 + \left(\frac{\Delta[\mathrm{L}]}{[\mathrm{L}]}\right)^2}$ (S8)

Inorganic mass of QDs:

The inorganic mass of the QDs (M_i) was determined by:

$$M_i = M - N_{\mathrm{L}} * (\text{molar mass of oleate}) - \frac{N_{\mathrm{L}}}{2} * (\text{atomic mass Cd}) \quad \text{(S9)}$$

Uncertainty: $\Delta M_i = \sqrt{(\Delta N_{\mathrm{L}} * 56.21)^2 + (\Delta N_{\mathrm{L}} * 282.46)^2 + (\Delta M)^2}$ (S10)

Units of CdSe:

The units of CdSe (U_{CdSe}) were determined from the inorganic mass of the QD using equation S7.

$$U_{\mathrm{CdSe}} = \frac{M_i}{\text{molar mass of CdSe}} \quad \text{(S11)}$$

Uncertainty: $\frac{\Delta U_{\mathrm{CdSe}}}{U_{\mathrm{CdSe}}} = \sqrt{\left(\frac{\Delta M_i}{M_i}\right)^2}$ (S12)

Volume of a CdSe QD:

The volume of a single CdSe QD was determined using the units of CdSe and the density of CdSe:

$$V_{\mathrm{QD}} = \frac{U_{\mathrm{CdSe}}}{\text{density of CdSe}} \quad \text{(S13)}$$

The density of CdSe is 18.3 units/nm^3.

Uncertainty: $\frac{\Delta V_{\mathrm{QD}}}{V_{\mathrm{QD}}} = \sqrt{\left(\frac{\Delta U_{CdSe}}{U_{CdSe}}\right)^2}$ (S14)

Diameter of CdSe:

The diameter (D) of the CdSe QDs was calculated as:

$$D = \sqrt[3]{\frac{(V*6)}{\pi}} \quad \text{(S15)}$$

Uncertainty: $\Delta D = \frac{1}{3}\frac{\Delta V_{\mathrm{QD}}}{V_{\mathrm{QD}}} D$ (S16)

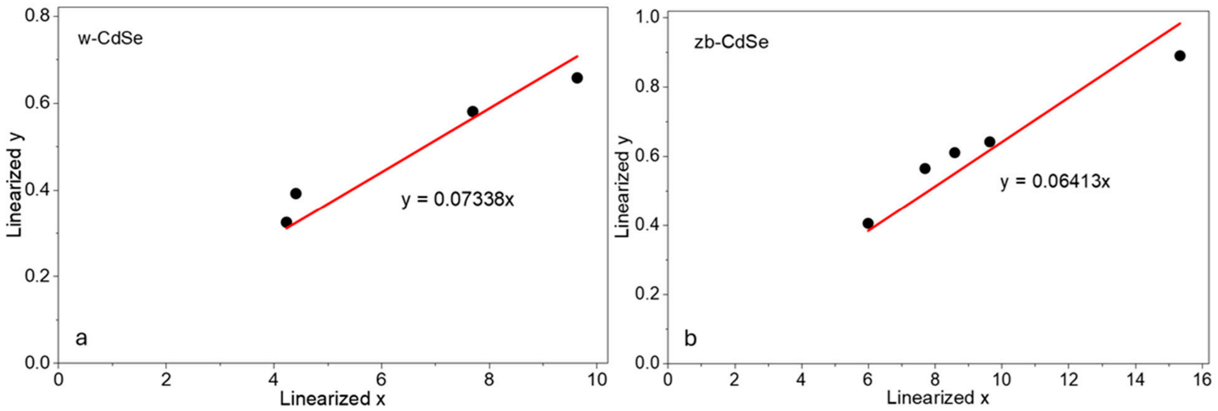

Figure S1. Linearized fits to the Hens[2] sizing equation of (a) wz-CdSe (b) zb-CdSe.

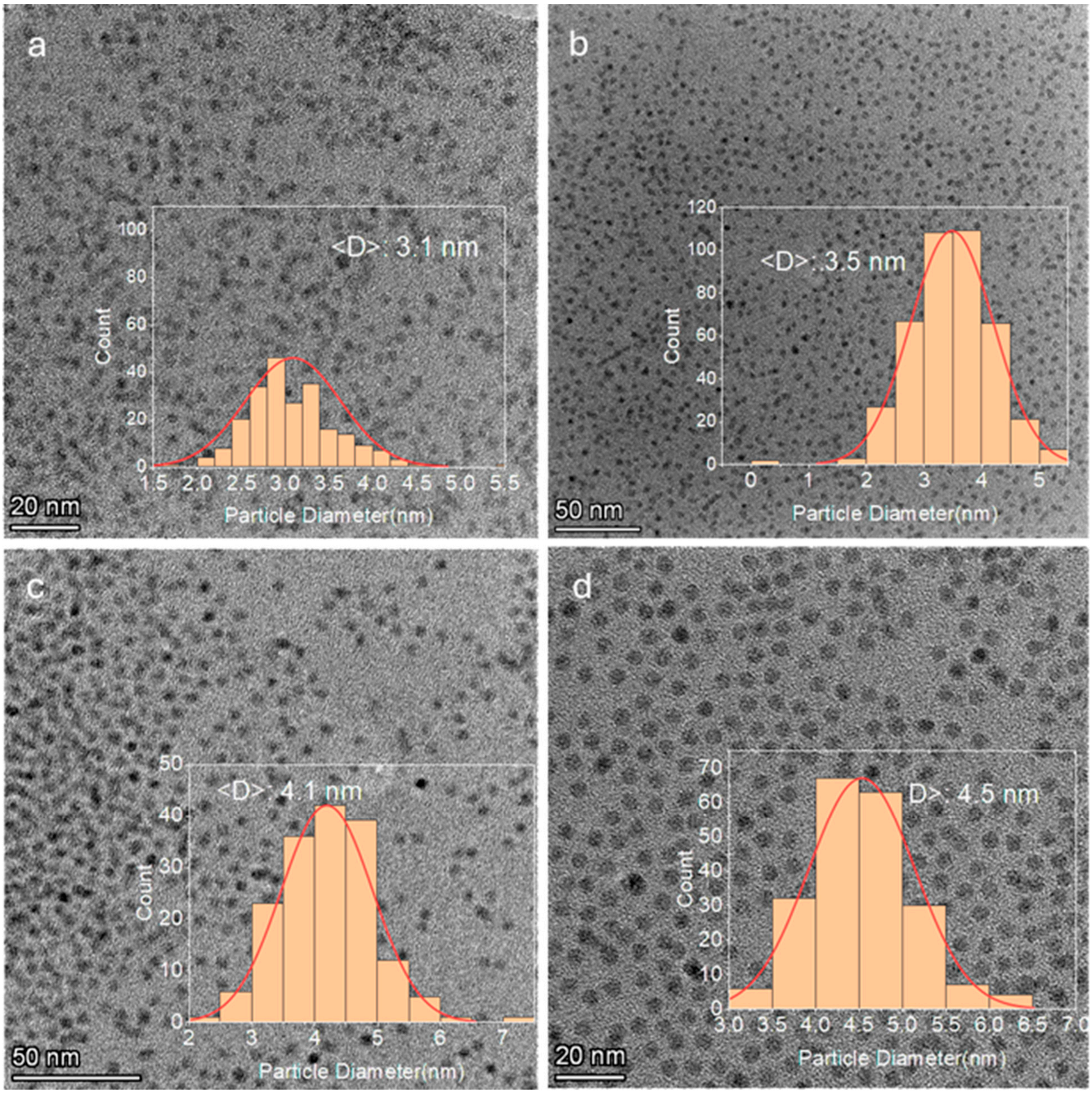

Figure S2. TEM Images of wz-CdSe QDs absorbing at (a) 2.27 eV, (b) 2.22 eV, (c) 2.09 eV, (d) 2.04 eV.

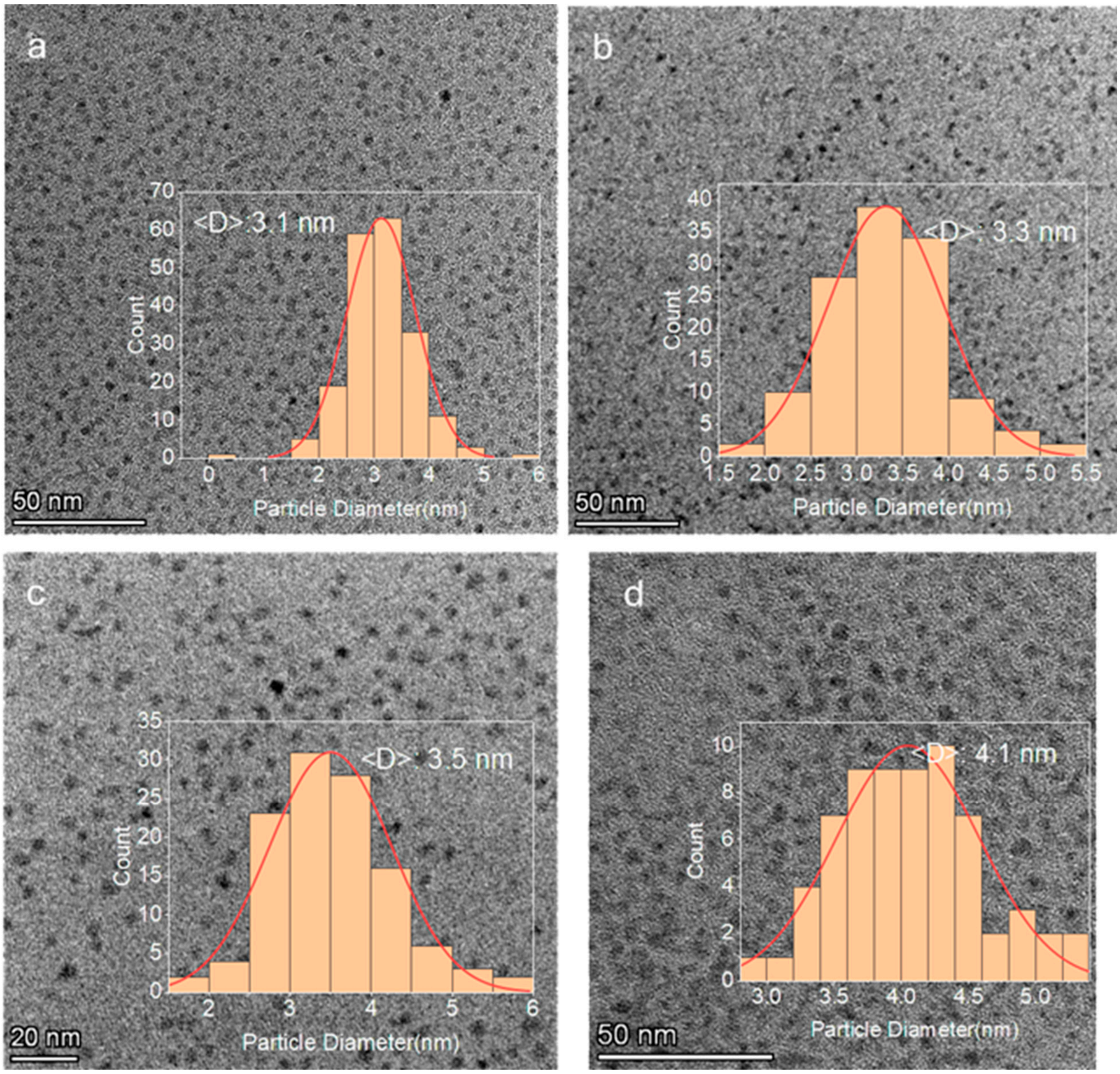

Figure S3. TEM Images of zb CdSe QDs absorbing at (a) 2.26 eV, (b) 2.24 eV, (c) 2.21 eV, (d) 2.10 eV

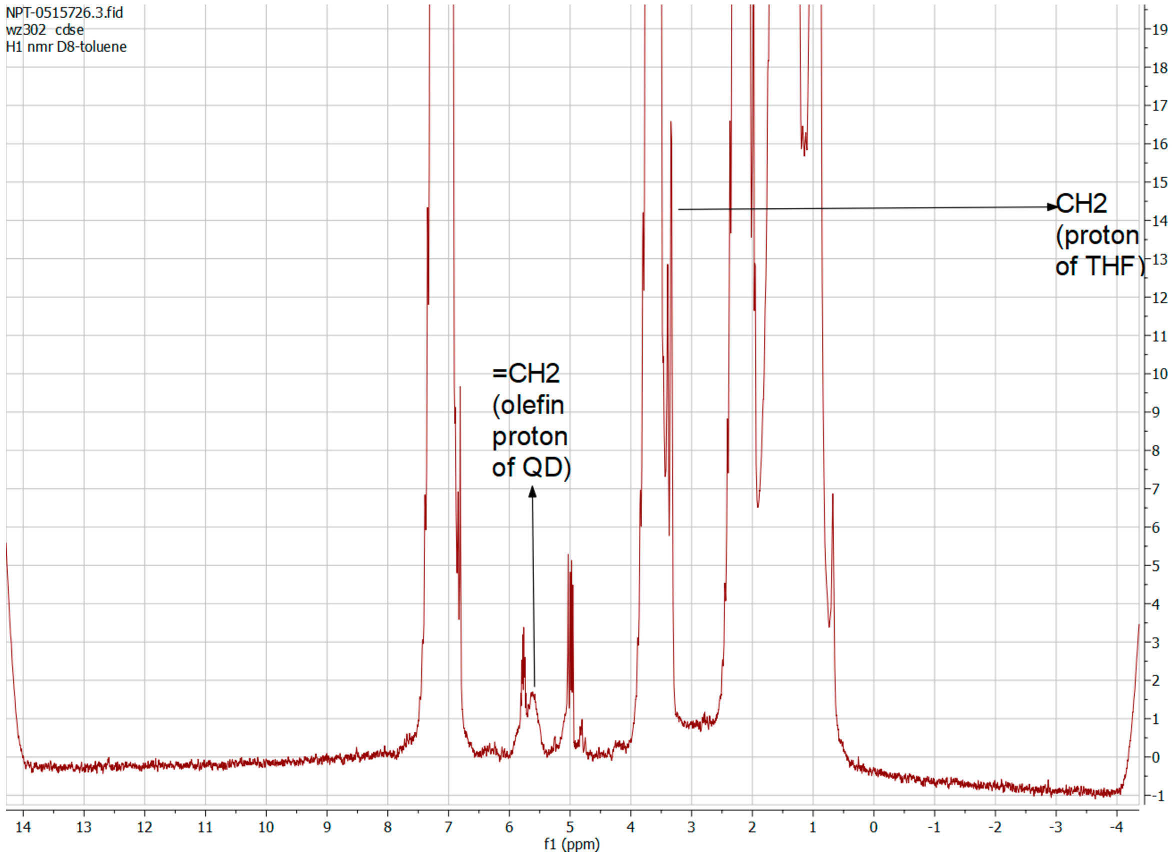

Figure S4. NMR of w-CdSe QDs with the internal standard THF.

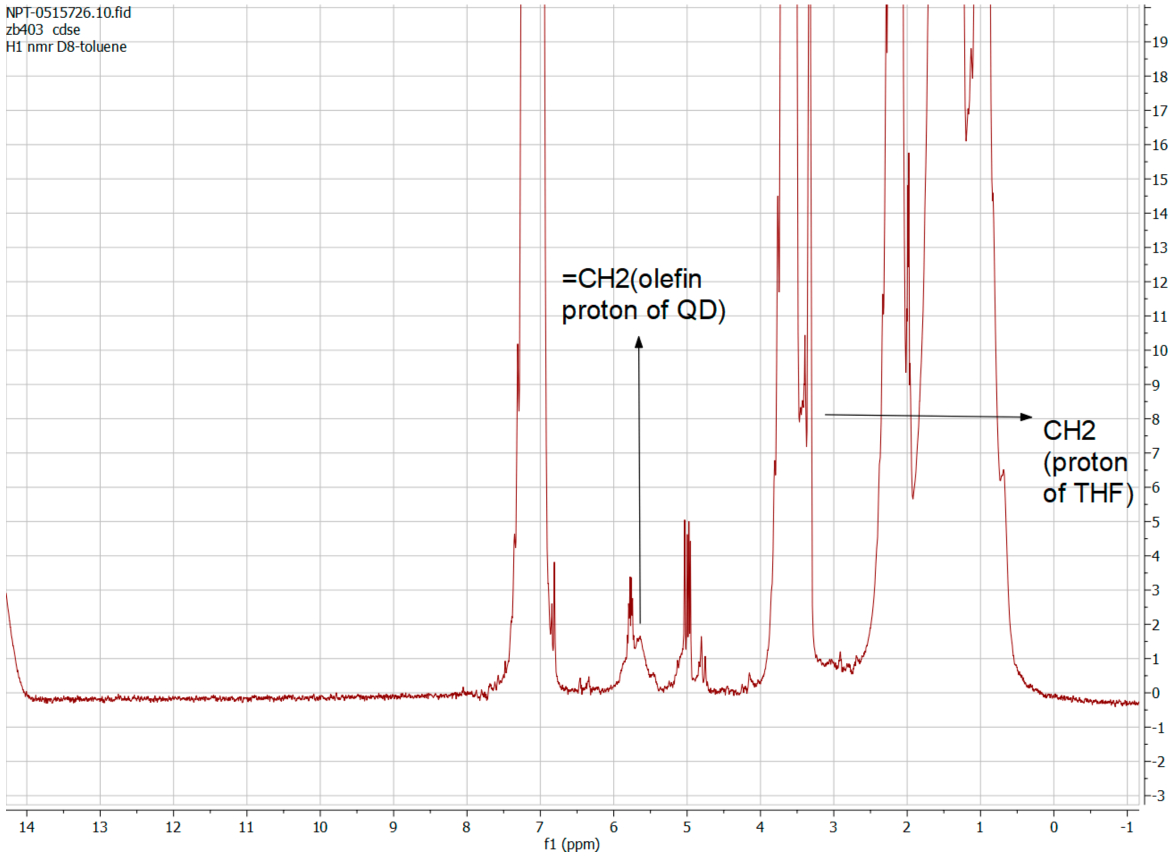

Figure S5. NMR of zb-CdSe QDs with internal standard THF.

Sample calculation of ligand concentration of QD [L] from NMR

[L]= $\frac{normalized\ area\ ligand * [THF\}}{normalized\ area\ THF}$

[THF]=0.103 M

Normalized area= $\frac{integrated\ area\ at\ chemical\ shift}{number\ of\ protons\ at\ chemical\ shift}$

Normalized area ligands= $\frac{2}{2}$ =1

Normalized area THF = $\frac{252.7591}{4}$ = 63.2

[L]= $\frac{1*[0.103]}{63.2}$ = 1.63 mM

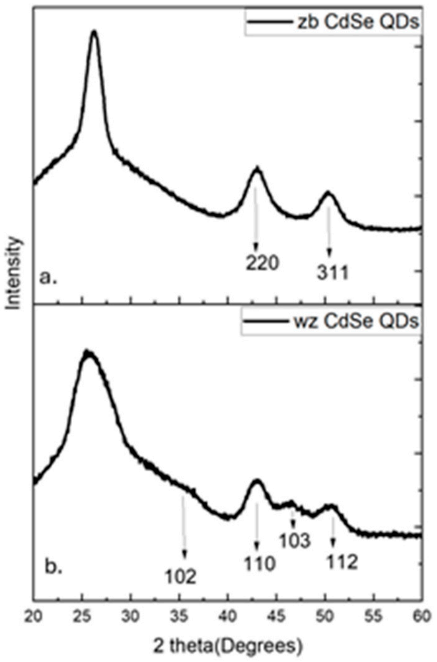

Figure S6. Powder XRD of (a) zb-CdSe QDs absorbing at 2.21eV and (b) wz-CdSe QDs absorbing at 2.09 eV with the corresponding Miller indices.

Table S1. Extinction coefficients of wurtzite (wz) and zinc blende (zb) CdSe QDs.

**Crystal Structure**	**Band Gap (eV)**	**Extinction Coefficient ($M^{-1}mm^{-1}$)**
wz	2.27	21048 ± 3133
wz	2.22	17408 ± 4431
wz	2.09	27505 ± 4471
wz	2.04	48318 ± 10492
zb	2.41	7730 ± 1269
zb	2.26	12762 ± 2475
zb	2.24	15276 ± 4638
zb	2.21	13986 ± 4853
zb	2.10	8320 ± 2547

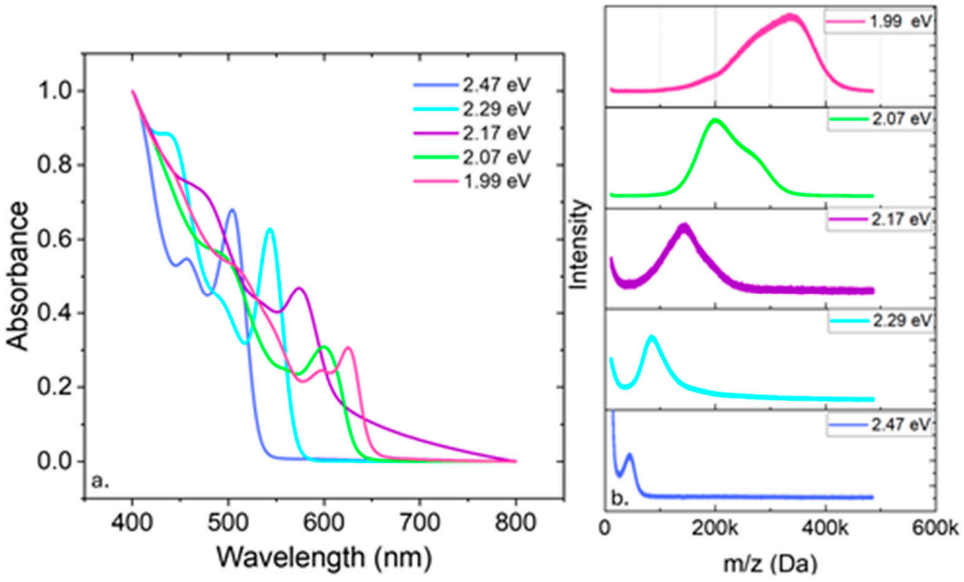

Figure S7. (a) Absorption spectra of five samples of myristate-capped zb-CdSe QDs of different absorption energies of the first exciton (1.99 – 2.47 eV). (b) The MALDI-TOF MS of the five samples of zb-CdSe QDs.

Table S2. A summary of the molecular weight from MALDI, the composition of myristate-capped zb-CdSe QDs, and the MALDI-MS size.

First-exciton absorption energy	Peak mass from MS	Deduced representative composition	Calculated size of the core
2.47 eV	42655 ± 14540 Da	$(CdSe)_{156\pm79}[Cd(C_{13}H_{27}CO_2)_2]_{23}$	2.6 ± 0.4 nm
2.29 eV	88452 ± 22306 Da	$(CdSe)_{379\pm119}[Cd(C_{13}H_{27}CO_2)_2]_{28}$	3.5 ± 0.4 nm
2.17 eV	143044 ± 35402 Da	$(CdSe)_{644\pm187}[Cd(C_{13}H_{27}CO_2)_2]_{35}$	4.1 ± 0.4 nm
2.07 eV	215646 ± 55302 Da	$(CdSe)_{910\pm295}[Cd(C_{13}H_{27}CO_2)_2]_{74}$	4.6 ± 0.5 nm
1.99 eV	316245 ± 68018 Da	$(CdSe)_{1293\pm364}[Cd(C_{13}H_{27}CO_2)_2]_{122}$	5.2 ± 0.5 nm

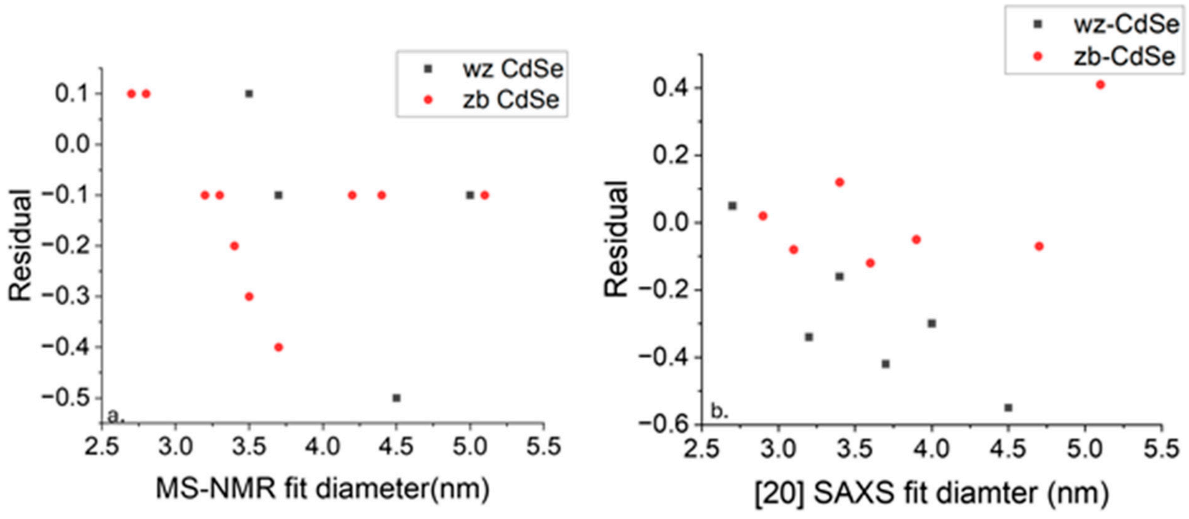

Figure S8. Residual plots of (a) MS-NMR fit and (b) SAXS fit for wz- and zb-CdSe QDs.

Table S3. Summarizing the χ^2 for the MALDI and SAXS fits for their respective data sets for wz and zb CdSe QDs.

**QDs**	**MS-NMR fit χ^2**	**[20] SAXS fit χ^2**
wz-CdSe	0.063115	0.181978
zb-CdSe	0.100672	0.045082

Table S4. Comparison of the Cd:Se ratio for wz-CdSe QDs determined by MALDI-MS and ICP-MS.

**First Exciton Absorption Energy (eV)**	**Cd:Se ratio determined by MS-NMR**	**Cd:Se ratio determined by ICP-MS**	**MS-NMR Projected Diameter (nm)**
2.27	1.05	1.46 ± 0.14	3.4 ± 0.2
2.22	1.05	1.61 ± 0.15	3.8 ± 0.4
2.09	1.01	1.48 ± 0.14	5.0 ± 0.3
2.04	1.02	1.44 ± 0.14	5.1 ± 0.4

Table S5. Comparison of the Cd:Se ratio for zb-CdSe QDs determined by MALDI and ICP-MS.

**First Exciton Absorption Energy (eV)**	**Cd:Se ratio determined by MS-NMR**	**Cd:Se ratio determined by ICP-MS**	**MS-NMR Projected Diameter (nm)**
2.41	1.15	2.18 ± 0.21	2.7 ± 0.2
2.26	1.08	1.94 ± 0.19	3.4 ± 0.3
2.24	1.10	1.66 ± 0.16	3.6 ± 0.5
2.21	1.08	1.63 ± 0.15	3.8 ± 0.6
2.10	1.03	1.62 ± 0.15	4.3 ± 0.5

For both crystal structures of Cd and Se, we observed a larger Cd:Se ratio in ICP-MS than from the combination of MALDI-MS and NMR. Part of the reason is the 10% uncertainty of ICP-MS, which we observed in control measurements of Se of known concentration. However, observed excess of the metal over Se is a known problem in ICP. Even when the best care is taken to digest CdSe QDs with nitric acid and most of it converts to water-soluble H_2SeO_3, part of it converts to colloidal Se, which is difficult to detect but it may aggregate and deposit in the ICP measurement line.

(1) Xie, L.; Shen, Y.; Franke, D.; Sebastián, V.; Bawendi, M. G.; Jensen, K. F. Characterization of Indium Phosphide Quantum Dot Growth Intermediates Using MALDI-TOF Mass Spectrometry. *Journal of the American Chemical Society* **2016**, *138* (41), 13469-13472. DOI: 10.1021/jacs.6b06468.
(2) Aubert, T.; Golovatenko, A. A.; Samoli, M.; Lermusiaux, L.; Zinn, T.; Abécassis, B.; Rodina, A. V.; Hens, Z. General Expression for the Size-Dependent Optical Properties of Quantum Dots. *Nano Letters* **2022**, *22* (4), 1778-1785. DOI: 10.1021/acs.nanolett.2c00056.
